# Assisted probe guidance in cardiac ultrasound: A review

**DOI:** 10.3389/fcvm.2023.1056055

**Published:** 2023-02-14

**Authors:** Sofia Ferraz, Miguel Coimbra, João Pedrosa

**Affiliations:** ^1^Institute for Systems and Computer Engineering, Technology and Science INESC TEC, Porto, Portugal; ^2^Faculty of Engineering of the University of Porto (FEUP), Porto, Portugal; ^3^Faculty of Sciences of the University of Porto (FCUP), Porto, Portugal

**Keywords:** echocardiography, machine learning, deep learning, image acquisition, ultrasound

## Abstract

Echocardiography is the most frequently used imaging modality in cardiology. However, its acquisition is affected by inter-observer variability and largely dependent on the operator’s experience. In this context, artificial intelligence techniques could reduce these variabilities and provide a user independent system. In recent years, machine learning (ML) algorithms have been used in echocardiography to automate echocardiographic acquisition. This review focuses on the state-of-the-art studies that use ML to automate tasks regarding the acquisition of echocardiograms, including quality assessment (QA), recognition of cardiac views and assisted probe guidance during the scanning process. The results indicate that performance of automated acquisition was overall good, but most studies lack variability in their datasets. From our comprehensive review, we believe automated acquisition has the potential not only to improve accuracy of diagnosis, but also help novice operators build expertise and facilitate point of care healthcare in medically underserved areas.

## 1. Introduction

Cardiovascular diseases (CVDs) are a leading cause of mortality worldwide and a major contributor to disability (1). Over the past few decades, significant advancements have been made in cardiovascular research and practice with the goal of improving the diagnosis and treatment of heart disorders as well as decreasing the mortality of CVD. Modern medical imaging methods are now widely utilized for cardiac diagnosis, illness monitoring, treatment planning, and prognosis. Examples include computed tomography, ultrasound (US), and magnetic resonance imaging (2).

Echocardiography in particular is the most often used non-invasive cardiac technique and is the imaging modality recommended by European Society of Cardiology for diagnostic and prognostic reasons of the majority of cardiac diseases (3). In the United States alone, 7.1 million echocardiograms are performed yearly, and approximately 20% of Medicare members receive at least one echocardiogram each year (4). In contrast with other imaging modalities, echocardiography is portable, has good temporal resolution, does not use ionizing radiation, and is inexpensive (3). Additionally, it is the only imaging technique that permits real-time imaging of the heart, allowing for the detection of multiple abnormalities instantly (5).

Nonetheless, echocardiographic analysis is associated with several challenges. Namely, lengthy procedures (usually longer than 20 min), multiple measurements that increase user subjectivity and duration, complex analysis during the echocardiographic assessment, high standards for individual evaluations, substantial operator subjectivity, and broad observational ranges and differences among observers that remain even under standardized circumstances. Due to these restrictions, there is high demand for medical specialists trained in the field of echocardiography (6). In this context, artificial intelligence (AI) can potentially reduce the inter observer variation and overcome the lack of extensive operator experience.

Although AI was first introduced in the 1950s, the application of AI in echocardiography is still in its infancy. AI techniques can be used to recognize a wide range of patterns within echocardiograms, since it can account for each pixel and their relationship, as well as associated clinical metadata (5). AI for echocardiograms can be utilized in cardiology, primary care, and emergency clinics for low-cost, serial, and automated evaluation of heart anatomy and function by experts and non-experts. Additionally, it would allow for the customized longitudinal monitoring and preliminary diagnosis of patients with cardiovascular risk factors and the triage of arriving patients with chest discomfort in an emergency unit (7). AI applications in echocardiography, based on machine learning (ML) or deep learning (DL) frameworks, have recently been proposed for several tasks but significant limitations remain, such as inadequate clinical model generalization, poor robustness, and inadequate standardization of echocardiography (6). Furthermore, DL algorithms in echocardiography have the added challenge of demanding big data (8). The lack of interpretability of AI solutions is also often considered a major limitation towards the clinical implementation of these tools. Finally, while the automation of tasks in echocardiography has received considerable attention in the medical imaging community, most studies have focused on problems related to echocardiogram interpretation, such as left ventricle (LV) segmentation, overlooking tasks related to acquisition such as quality assessment (QA), cardiac view classification (CVC) and probe guidance. However, the automation of echocardiogram acquisition is of great value as it would offer standardization and open doors to less experienced US users, which could have a significant impact in disadvantaged settings.

In this review, we provide an overview of the current state of AI in echocardiography acquisition and the challenges that are being confronted. In recent years, there have been several review papers that presented overviews about applications of DL-based methods for US analysis. However, to our knowledge, none of them has provided a systematic overview focused on automated echocardiography acquisition. As such, this review paper aims at providing a comprehensive overview of the state-of-the-art of DL algorithms applied to echocardiography acquisition tasks, such as assessment/enhancement of image quality, CVC and assisted probe guidance (Section 3). Further, this paper analyzes the various challenges of incorporating AI in the clinical workflow and provides insight into the future of automated echocardiography (Section 4).

## 2. Review methodology

A thorough review of the literature was made using the Google Scholar and PubMed search engines. Initially, the terms “Machine Learning OR Deep Learning” along with “Echocardiography OR Echocardiogram” were used to search relevant articles. Search filters were then added with the inclusion of terms “quality,” “view classification” and “guidance OR assisted acquisition” to refine the search.

**Figure 1:**
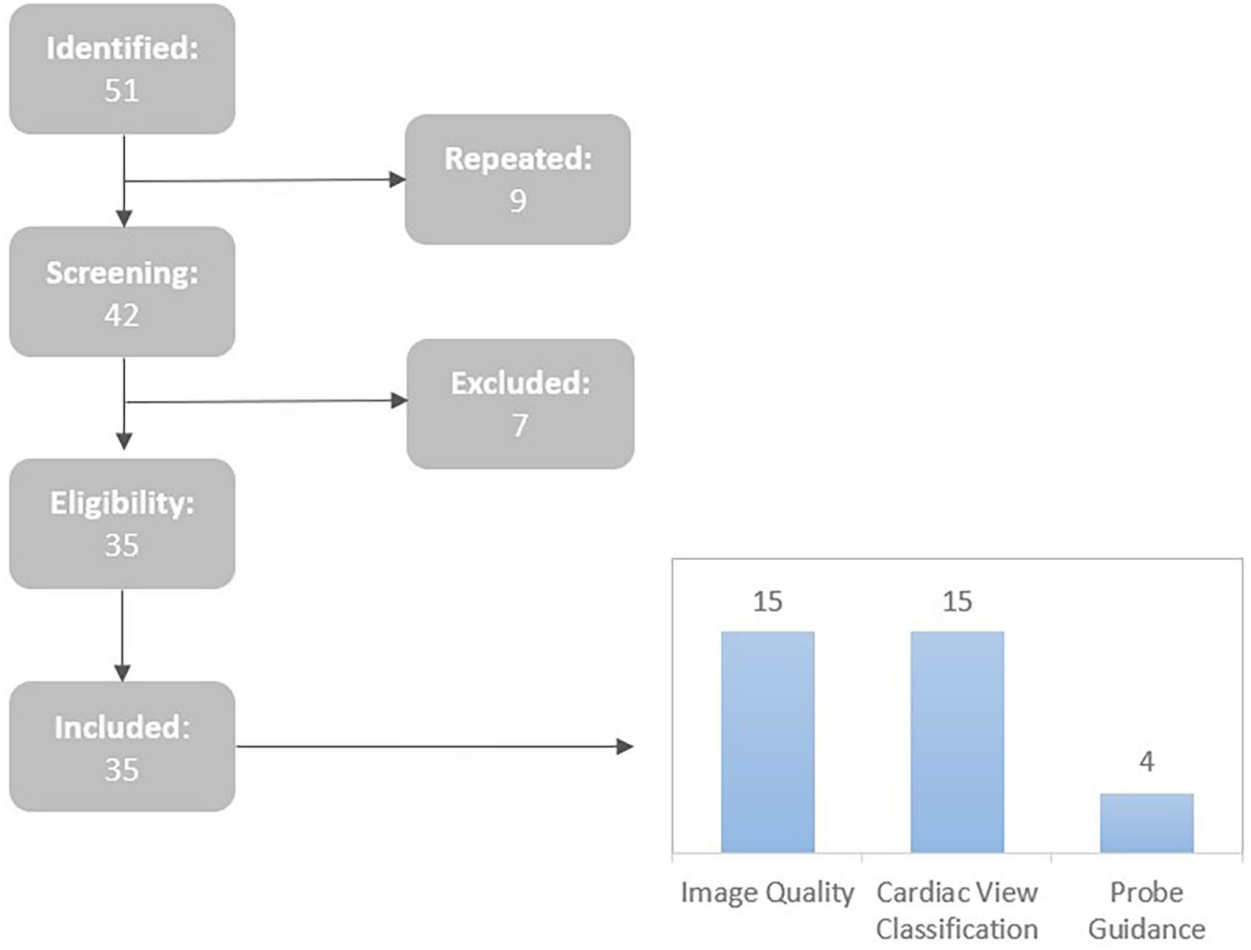
Summary of the article selection process and sorting of the included articles by acquisition tasks.

Peer-reviewed journal publications were included as well as conference proceedings in this field that describe the application of ML to echocardiographic images, before June 2022. A total of 35 papers that are relevant to the scope of this review (see Figure 1) were included. Furthermore, the reports were divided into three groups based on the acquisition task performed: assessment/improvement of echocardiographic quality, view classification and assisted probe guidance (see Figure 1 for the included articles under each group). It’s also worth mentioning that, although the main findings of this review focus on transthoracic echocardiograms (TTE), some studies on Fetal and Doppler cardiac USwere also included.

The article selection was based on the following inclusion (I) and exclusion (E) criteria: (I1) Articles that used AI techniques regarding echocardiography acquisition; (I2) Articles that were complete and written in English; (I3) Articles published since 2017; (E1) Articles that did not use 2D echocardiographic data; (E2) Articles that were incomplete or were abstracts.

A total of 51 publications were found during searches in the top scientific databases. Seven were eliminated after reading the abstracts and titles. Thus, 35 items remained. None of the articles passed the exclusion criteria (E1 and E2) after being read in their entirety, hence they were all included in the review.

## 3. Artificial intelligence applications for echocardiography acquisition

As previously discussed, AI and ML/DL are playing increasingly important roles in echocardiography, as they have been shown to facilitate multiple steps along the clinical care workflow and allow for a higher reproducibility. The process of echocardiographic AI acquisition involves several steps performed during each examination. When developing the application of AI, it is necessary to divide these steps into separate tasks. In this section, we provide a summary of machine learning based applications for the three main echocardiographic tasks regarding acquisition: (1) assessment/improvement in image quality; (2) classification of the cardiac window; and (3) assisted US probe guidance.

### 3.1. Image quality

Echocardiography quality can be described as a parameter that directly corresponds to the visibility of targeted anatomical structures, landmarks, and boundaries. The US equipment settings and the acoustic qualities of the patient have a significant impact on the quality of the acquired echocardiogram (9). Furthermore, the quality of an echocardiogram depends heavily on the sonographer’s skill and objectivity in obtaining the most optimal intersection of the imaging plane with the heart structure. While competent operators, such as imaging technicians and cardiologists, are capable of discovering the optimum acoustic window resulting in high-quality images, less-experienced users are more likely to acquire data with suboptimal image quality (10).

**Figure 2:**
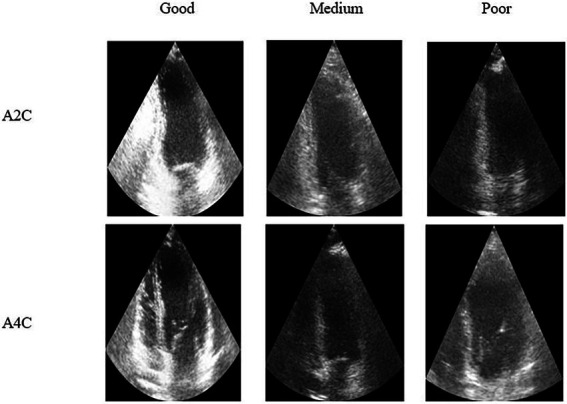
Representative echocardiography images, across two different cardiac views, from good to poor quality ultrasound [images from (12)].

Accurate diagnosis can be significantly impacted by poor quality echocardiographic imagescaptured by inexperienced ultrasonography operators (11) (Figure 2). In fact, clinical metrics are more reproducible in high-quality echocardiogram data, whereas even experienced cardiologists may find it difficult to evaluate low-quality scans (9). As a result, poor image quality can impact cardiac chamber segmentation, leading to a misclassification of the patient’s treatment requirements (10). Several studies have made significant attempts to develop automatic systems that can provide real-time image quality feedback to the operator and thus aid the sonographer in obtaining optimal quality views. The areas of research regarding echocardiogram quality mainly focus on QA, quality control and quality enhancement.

#### 3.1.1. Quality assessment

Image quality assessment is currently a subjective procedure in which an echocardiography specialist visually inspects and scores an image based on certain features. Since this step is performed by a qualified professional, it has certain shortcomings, such as reliance on the operator’s experience, substantial workload, and high inter- and intra-observer variability (13). The subjectivity associated with this process may result in inconsistency in diagnostic interpretation and decision-making (11). As a result, an automated and objective quantitative technique for assessing image quality would be beneficial to research and clinical practice (14).

Most recently proposed methods to estimate US image quality are based on DL (Table 1). For instance, Abdi et al. (10) aimed to automatically estimate a score of echocardiogram quality (between 0 and 5) for operator feedback in apical 4 chamber (A4C) view. To achieve this, the authors used Particle Swarm Optimization (PSO) and a custom deep convolutional neural network (CNN), and reported an absolute error (0.71 ± 0.58) comparable to the measured intra-rater reliability. However, the evaluation was limited to end-systolic frames and the proposed method did not make use of the data presented in subsequent echocardiography frames. As a result, the authors expanded on their earlier research (15) and developed a DL model for QA and used 2,450 echocardiography cine loops over 5 standard imaging views. The researchers achieved an accuracy rate of 85% for quality scores on 20% of the dataset. With the rise of Point- of- Care Ultrasound (POCUS) technology, mobile compatible solutions for quality estimation are more present than ever. In this context, Van Woudenberg et al. (17) proposed an Android application to provide the user with real-time feedback of both CVC and image quality. The authors used a single DL network with a DenseNet model and Long Short Term Memory (LSTM) features, trained on a dataset of over 16,000 echocardiogram cines distributed across the 14 cardiac views. While the authors report the models speed and latency (30 frames per second and 352.91 ± 38.27 ms, respectively), the evaluation metrics of the model were not shared in this paper. Nonetheless, the system is compatible with Android mobile device and could be used together with POCUS.

**Table 1:** Deep learning based studies for quality assessment.

Authors	Year	No. views	DL model	Dataset	Performance
Abdi et al. (10)	2017	1	PSO + DCNN	6,916 end-systolic echocardiography images	MAE 0.71 ± 0.58
Abdi et al. (15)	2017	5	VGG + LSTM	2,450 echo cines	Acc 85%
Liao et al. (11)	2019	14	DenseNet + LSTM	14,443 echocardiography studies from 3,157 unique patients	MAE 0.09 ± 0.08
Luong et al. (16)	2020	9	DenseNet + LSTM	14,086 echo video clips	MAE 0.12 ± 0.09
Van Woudenberg et al. (17)	2018	14	DenseNet + LSTM	16,000 echo cines	----
Zamzmi et al. (18)	2019	----	VGG-16 and ResNet-50	100 patients	Acc 88.9%

MAE, mean absolute error; Acc, accuracy.

On the other hand, Luong et al. (16) aimed to automatically assess the quality score of TTEs in hospitalized patients across 3 clinical groups: mechanically ventilated patients and two matched spontaneously breathing controls. For this purpose, the authors used the model previously published in (17), and used 16,772 2D TTE videos. The overall estimated maximum quality score was significantly poorer for mechanically ventilated TTEs (0.55) compared with either control group (Control 1: 0.64, Control 2: 0.61). The authors also sought to investigate the relation between image quality and completeness of TTE reporting (i.e., the proportion of standard parameters documented), finding that lower quality TTEs were associated with fewer reported parameters.

While existing automated methods for QA focus on conventional echocardiography, Zamzmi et al. (18) proposed the first study for automatically detecting and classifying different blood flows in Doppler imaging, as well as assessing their quality. The authors achieved an overall accuracy of 88.9% for flow QA. Later, in (19), they used a different approach in TTE by developing a lightweight model (MobileNetV2-s) for retrieving echocardiograms of acceptable quality, thus automating the process of excluding low-quality echocardiograms performed by echocardiographers in clinical practice. Prior to training, the authors used self-supervised representation to learn low-level features. The performance of the QA algorithm was compared with VGG16 and ResNet18 models. The proposed approach outperformed the state-of the-art models and achieved a fast inference speed. Interestingly, Huang et al. (20) developed a QA method by quantifying echocardiographic video features, with the goal of improving precision in strain measurements. For this purpose, the authors first trained a CVC model (DenseNet-121) and assigned the quality score based on the aggregation of discriminative features learned by the convolution process (i.e., the more extracted features an input image has, the higher the quality). Their results suggest that this quality metric could serve as a quality index for assessing the reliability of strain values, for a specific cardiac view.

Despite recent progress in automating QA, the accuracy of deep neural networks may be directly impacted by the uncertainty of labels in clinical data resulting from the mapping of an expert’s assessment of quality to the echocardiogram image. In fact, the accuracy of the quality quantification might be impacted by observer variability in the expert’s evaluation (11). In order to model label uncertainty in data, Liao et al. (11) suggested a new method and showed that this modeling approach outperforms traditional regression approaches by 5.7%.

QA has the potential of transforming the training landscape for novice operators, as well as supporting more experienced scanners. However, there are still some challenges to overcome in this field of research, namely the lack of variability in the datasets used, which are often composed of single center and/or single vendor ultrasounds and annotated by one or few professionals. Further work should employ data from multiple sites using a variety of US vendors, and preferably annotated by several qualified experts to improve the inter- and intra- variability, respectively. Another common problem among some authors is the low number of cardiac views present in the dataset, restricting the usage of the model to one or few cardiac views. Thus, future research should attempt to incorporate a higher number of echocardiographic windows in the dataset.

As shown on Table 1, most approaches for automatic QA consist on the application of CNN to extract relevant features and LSTMlayer to extract the temporal information across echocardiographic frames. The most common employed CNN architecture in the reviewed papers is the Dense Convolutional Network (DenseNet), followed closely by the Visual Geometry Group (VGG) model. Although QA systems have their utility in clinical practice, these models only predict an overall image quality score and offer no clue as to why the image is being tagged as low quality or how to improve it to obtain optimal images. As such, future work could be improved by new strategies, such as quality control, that offer data regarding valve structure, image depth and gain, and further support the operator.

#### 3.1.2. Quality control

Contrary to quality assessment, quality control (QC) offers a symbolic score to represent quality along with a more detailed report depicting quality attributes (e.g., gain, on-axis imaging), thereby ensuring a more interpretable model (14). Dong et al. (13) focused on real-time QCof fetal US cardiac 4 chamber views, by proposing a CNN-based framework to evaluate important imaging properties (e.g., gain and zoom) and the presence of key anatomical structures. The authors implemented 3 different networks: a basic CNN to classify the 4 chamber views in the raw echocardiographic dataset; a deeper CNN to determine the gain and zoom of the US; and an aggregated residual visual block net to detect the anatomical structures on the image. The highest mean average precision achieved by the author’s framework was of 93.52% at a speed of 101 frames per second.

Smistad et al. (21) used a different method to measure image quality by employing 5 categories determining the criteria for a high-quality image, which included the lack of foreshortening. Apical foreshortening occurs when the US probe is positioned improperly by the operator and the imaging plane does not cut through the actual apex of the LV, leading to erroneous volume and, consequently, EF assessments. To specifically identify foreshortening, the author’s method employs segmented images from both A4C and apical two chamber (A2C) views at both the ED and ES time periods of the cardiac cycle. Similarly, Labs et al. (14) developed a hybrid model with CNN and LSTM layers to automatically rate the quality of the A4C view based on LV foreshortening, as well as other quality parameters, such as on-axis imaging and contrast/gain. The authors achieved an average accuracy of 86% on the test dataset, using 1,039 echocardiographic patient datasets labeled by a professional cardiologist for model development and testing.

QC for the acquired US images would enrich quality classification systems and provide interpretability of the models. However, QC requires a more resource intensive annotation process, which can lead to more variability among annotators than a scoring system. To date, the number of articles published on QC is scarce, which may be due to the high volume of annotations required for these frameworks.

#### 3.1.3. Quality enhancement

In addition to echocardiogram quality assessment and quality control, several groups have tackled medical image enhancement using DL. Jafari et al. (22) proposed generative adversarial networks (GANs), more specifically an anatomically constrained CycleGAN, to improve echocardiography quality in A4C view, for the purpose of LV segmentation. Their findings demonstrated that the suggested strategy increases the LV segmentation’s robustness, with the worst-case Dice score rising by 15% over the baseline. Later, using a fully convolutional deep translation model, the same group (9) sought to translate POCUS images to the superior quality of high-end cart-based US systems. The deep transfer model was once more trained using a constrained cycle-consistent GAN, and the suggested technique increased the accuracy of LV segmentation in apical cardiac view.

Similarly, Liao et al. (23) proposed an echocardiography image quality transfer network that can translate echocardiographic images towards a user-defined quality level using a multi-domain transfer approach known as StarGAN. The proposed quality transfer StarGAN utilizes the temporal information of echocardiogram images during the training phase and does not require pairs of low- and high-quality echocardiogram images. The authors demonstrated the effectiveness of the quality transfer by testing a CVC algorithm, and attaining a significantly improved classification accuracy. Furthermore, Diller et al. (24) examined the efficiency of DL algorithms for denoising TTE A4C images and eliminating acoustic shadowing artifacts, particularly in patients with congenital heart disease (CHD). A comparison was made between DL algorithms created on CHD samples and models trained only on structurally normal hearts. The models trained on congenital heart samples performed substantially better when exposed to instances from CHD patients, and the suggested network significantly improved image quality across diagnostic subgroups (*p* < 0.005 for all).

Quality enhancement techniques are essential in clinical practice, especially with the rise of POCUS imaging and the hardware limitations in compact pocket-sized US probes that result in low quality echocardiograms. Among the included studies, GANs are the preferential method for quality enhancement. It’s also important to recognize that quality enhancement is not an independent step but an essential stage for the analysis of echocardiographic images and it can greatly impact measurement or interpretation of the US.

### 3.2. View classification

**Figure 3:**
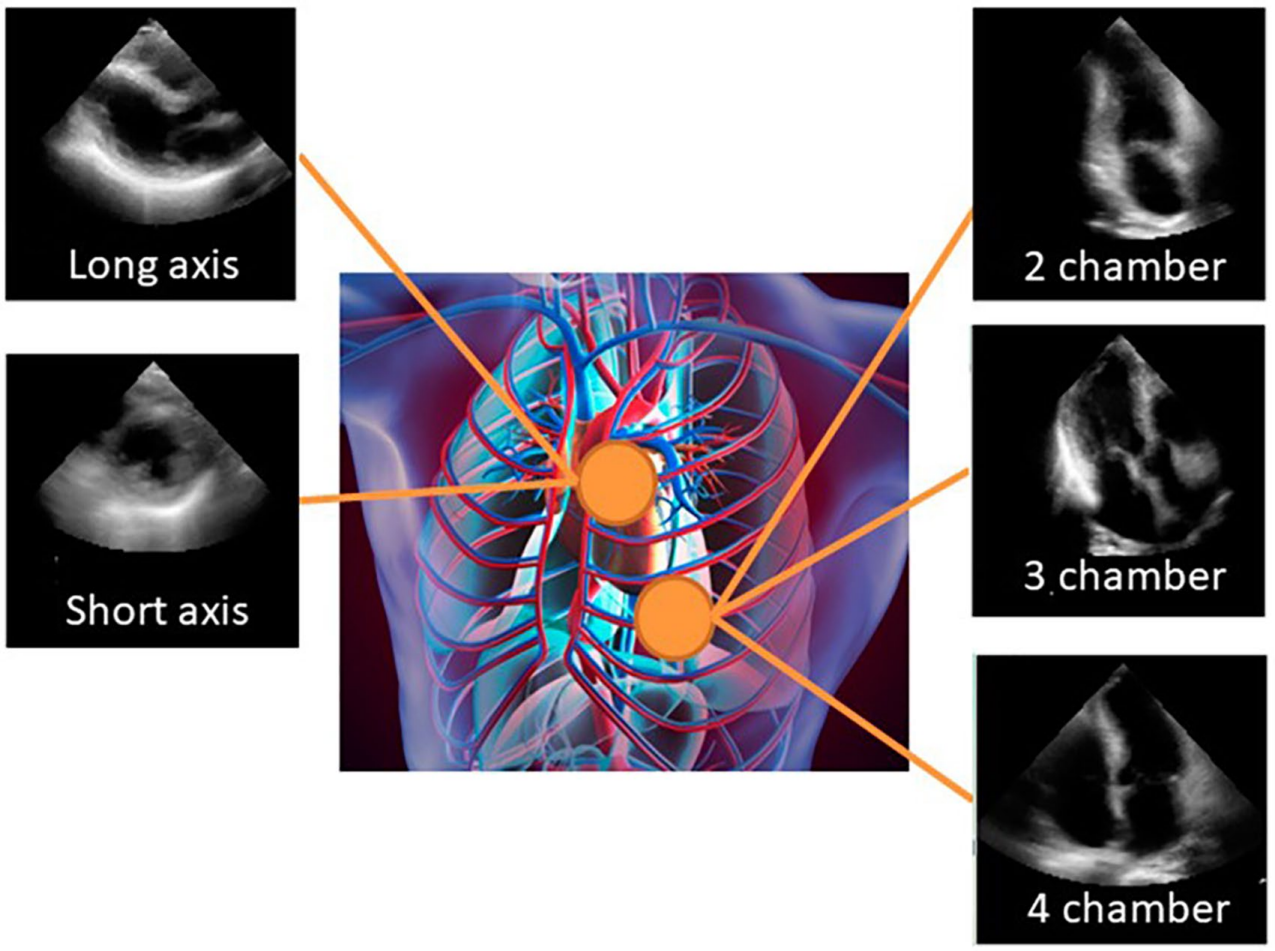
Sample of typical cardiac views: apical 2-chamber, apical 4-chamber, apical 3-chamber, parasternal long axis (PLAX), and parasternal short axis [PSAX; image from (28)].

Similarly to other cardiac imaging modalities, echocardiography studies need several viewpoints of the heart structures (25). Although an unlimited number of various views are theoretically feasible, 27 views have been recognized as the views to be obtained during a full TTE evaluation (26). Additionally, sonographers purposefully focus on substructures within an image, providing a variety of different perspectives by rotating and adjusting the US probe’s zoom level (25). Despite the multitude of different cardiac views, clinicians frequently use six standard views in a routine cardiac examination to assess the structure and function of the heart: the A4C, A2C, apical three chamber (A3C), parasternal short-axis mitral valve, parasternal short-axis papillary muscle, and parasternal short-axis apex (27) (Figure 3). These images record both spatial and temporal discriminative information, and allow for the identification, measurement and examination of several relevant anatomical structures, such as the LV (29).

Cardiac view classification consists of determining the image plane through the heart, and is an important first step in analyzing an echocardiogram (30, 31). This is a tedious, manual process that involves specialized training and is subject to both inter- and intra-observer variability (32). Given that the European Association of Echocardiography advises a minimum of 350 tests to obtain basic competency for standard TTE (30), finding accurate cardiac images can be particularly challenging for less experienced operators (30). Additionally, due to the echocardiography views’ noise and similar shape information, it may be difficult and tiresome to annotate huge databases, which could result in inaccurate or erroneous analysis (33).

As a result, reliable automatic classification of heart views offers several potential clinical uses, including workflow improvement, user guidance, a reduction in inter-user discrepancy, and increased accuracy for high throughput of echocardiographic data and subsequent diagnosis (32). Furthermore, by improving the automatic extraction of pertinent 2-D image planes from volumes, such a solution could improve user experience in 3-D US acquisitions. It can also supplement patient database archives by automatically labeling recordings, improving search functionality as well as data mining and categorization tools. In addition to the need for specialized resources, real-time CVC based pedagogic tools may be able to provide standardization through active quality assurance and assistance on probe alignment (30).

Automated image analysis has improved in accuracy and speed thanks to the use of ML techniques in computer vision. Previous attempts to classify cardiac views using ML (27, 34) were limited by the inability to distinguish between more than a few views at once, the use of only “textbook-quality” images for training, the low accuracy, or the relation to a single equipment vendor. These drawbacks made these methods unsuitable for widespread use. When it comes to complicated, high-dimensional issues like image recognition, the versatility of DL training represents a substantial advantage over prior ML methods and opens more opportunities for automating cardiac view (35).

For instance, Kusunose et al. (28) trained a CNN to classify 5 different echocardiographic views in a dataset of 17,000 labeled images. The researchers used 5-fold cross validation to evaluate the model performance, and the model performed the best in classifying video views in the independent cohort with an overall test accuracy of 98.1%. Similarly, Ostvik et al. (30) developed a real-time CVC using CNNs, considering up to 7 of the most common cardiac views. The authors used a dataset of 41,450 images from 460 videos and performed a 10-fold patient-based cross-validation. Their network surpassed the performance of other architectures (AlexNet and Inception), with accuracies of 98.3 and 98.9% on single frames and sequences, respectively.

Madani et al. (35) suggested a CNN based on labeled still photos and videos from 267 TTEs to simultaneously classify 15 standard views (12 video, 3 still). The model correctly identified 12 different video views with an overall test accuracy of 97.8% and, on still images drawn from 15 views, the model achieved an overall accuracy of 91.7%, in contrast to board-certified echocardiographers’ accuracy of 79.4% Later, the authors expanded on their work (31) by testing the impact of view segmentation prior to the classification task, and explored semi-supervised GANs to leverage learning from both labeled and unlabeled data. The segmentation pipeline shows an improvement of 1.59% in overall test accuracy when compared to the initial CNN model, and the semi-supervised GANs showed a potential for scenarios with large sets of unlabeled data (accuracy of 80% with less than 4% data).

On the other hand, Howard et al. (36) aimed at improving the current state-of-the-art of view classification by exploring the efficacy of time-distributed networks and two stream networks. The dataset used, from Imperial College Healthcare NHS Trust’s echocardiogram database, consists of 8,732 videos classified as one of 14 views by an expert. They show that these architectures reduce traditional CNN error rates from 8.1 to 3.9% by more than a factor of two. Additionally, they demonstrate that there is a similar pattern of discordance amongst views and that these networks’ accuracy is approaching expert agreement (3.6% discordance). Azarmehr et al. (32) used the same database to automatically identify 14 echocardiographic views through CNNs. The authors used neural architecture search to automate the human process of creating network topologies with much fewer trainable parameters and equal accuracy. The presented models could be employed for real-time detection of the typical echocardiographic views since they outperformed standard classification CNN architectures in terms of speed and classification performance (accuracy 88.4 to 96%, respectively). In (37), the authors developed a RetinaNet-based method for identifying 3 cardiac views and simultaneous detecting the LV. The dataset was collected from 2 hospitals in China and then augmented, resulting in a total of 1,238, 1,011, 404 images of the A4C, A2C and A3C view, respectively. For each view, the dataset was divided into a training set, a validation set, and an independent testing set using the ratio of 7: 1: 2. Their results show a classification accuracy of 1.00, 0.94, and 0.99 for the A2C, A3C, and A4C view, respectively. Additionally, Gao et al. (29) proposed a fused CNN architecture that incorporates both spatial and temporal information sustained by the video images of the beating heart. This design uses hand-crafted features within a data-driven learning framework. A collection of 432 image sequences collected from 93 patients was used. The best classification results for 8 viewpoint categories of echocardiographic recordings by this architecture were a 92.1% accuracy rate, compared to 89.5% when only one spatial CNN network is used.

To accommodate real-time POCUS solutions, Vaseli et al. (38) introduced a lightweight deep learning model based on knowledge distillation of 3 popular state-of-the-art architectures, VGG-16, DenseNet, and Resnet, for classification of 12 echocardiogram views. A collection of 16,612 echo cines from 3,151 different patients was used to create and evaluate their networks, achieving an accuracy of 88.1%.

Since CNNs developed in general cohorts may underperform in the setting of altered cardiac anatomy, Wegner et al. (25) aimed to classify 17 cardiac views in datasets depicting structural and congenital heart disease (C/SHD). For this purpose, the authors proposed a non-congenital CVC model trained on 14,035 echocardiograms, and a CNN trained and tested on 139,910 and 35,614 frames from patients with C/SHD, respectively. The non-congenital model performed worse in patients with C/SHD when compared to patients without cardiac disease (accuracy of 48.3 and 66.7%, respectively). In contrast, the model trained and tested specifically in patients with C/SHD achieved an accuracy of 76.1% in detecting the correct echocardiographic view, highligthing the importance of specific datasets. More recently, Zamzmi et al. (39) proposed an open world active learning framework for CVC, more specifically, the proposed network classifies images of known views into their respective classes and identifies images of unknown views. Then, a clustering approach is used to cluster the unknown views into various groups to be labeled by cardiologists, and consequently added to the initial set of known views thus updating the classification network. The authors developed a VGG-based autoencoder and trained it to learn echocardiographic features. Their framework was built and evaluated on the publicly available EchoNet-Dynamic dataset and a private dataset. Their findings corroborate that the performance of open world classifiers is higher than a traditional closed world classifier, in addition to significantly increasing the efficiency of data labeling and the robustness of the classifier.

Some authors attempted to develop a fully automated pipeline for echocardiographic interpretation, including the view classification task. For instance, Smistad et al. (21) reported a QA (refer to Section 3.1.1.) and view classification network as a step to ultimately measure LV volume and ejection fraction. The authors used the CVC network of Ostvik et al. to recognize 8different cardiac views. The view classification network was trained using 2-D US recordings from multiple views of 500 patients and evaluated in a dataset of 100 patients. Likewise, Zhang et al. (26) proposed a CNN to automatically identify 23 viewpoints as part of fully automated pipeline. The training data consisted of 7,168 individually labeled videos, and a 5-fold cross-validation was used to assess accuracy. The overall accuracy of their model was 84% at an individual image level.

Regarding Doppler imaging, Akkus et al. (40) presented a fully automated pipeline for mitral inflow Doppler analysis using 5 well-known CNN architectures to classify echocardiographic studies into 24 classes. The authors used a training and test dataset of 5,544 and 1,737 still images, respectively. The model performed with an overall accuracy of 97% in the test dataset. Similarly, to discriminate between 15 different echo perspectives, Zamzmi et al. (18) created a deep learning-based technique for Doppler flow categorization. They obtained overall accuracy of 91.6% using their flow classification network, which took inspiration from the well-known VGG.

View identification is one of the most important steps of a fully automated echocardiography analysis pipeline. As shown in Table 2, there is an accuracy range of 80–99% for a varied number of views. In some studies, customized CNN models were used and outperformed state-of-the-art CNN models. However, most of the datasets used were considerably small for DL applications, in addition to often being from a single center and/or single vendor.

**Table 2:** Deep learning based studies for cardiac view classification.

Authors	Year	No. views	DL model	Data/validation	Performance
Akkus et al. (40)	2020	24	Inception, ResNet50, Densenet, inception_resnet, VGG16	Training: 5544 still images of 140 patients; Testing: 1737 still images of 40 patients	Acc 97%
Azarmehr et al. (32)	2021	14	DARTS	8,732 videos of 374 patients; Training/Validation/Testing Ratio → 60:20:20	Acc 88.4 to 96%
Gao et al. (29)	2016	8	Fused CNN	Train: 280 videos Testing: 152	Acc 92.1%
Howard et al. (36)	2020	14	Customized CNNs	9,098 echocardiographic videos; Train/Testing Ratio: 75:25	----
Kusunose et al. (28)	2020	5	Customized CNNs	Training/Validation: 13,600 labeled images Testing: 3,400 views	Acc 98.1%
Madani et al. (31)	2018	15	3 CNN models	Training: 325980 images Testing: 21746 images	Acc 94.4% for the supervised model/ Acc 80% for the semi-supervised model
Madani et al. (35)	2018	15	Modified VGG-16	Training/Validation: 200,000 images (240 studies) Testing: 20,000 images (27 studies)	Acc 97.8% on videos; 91.7% on still images/AUC 0.996
Ostvik et al. (30)	2018	7	AlexNet, Inception, Proposed Model	Training/ Validation: 4582 videos (205 patients) Testing: 2559 videos (265 subjects)	Acc 98.3% on single frames; 98.9% on sequences
Smistad et al. (21)	2020	8	Ostvik et al. proposed network	Training/Validation: 500 patients Testing: 100 patients	----
Vaseli et al. (38)	2019	12	VGG-16, DenseNet, and Resnet	16,612 echo cines obtained from 3,151 unique patients Training/Validation/Testing Ratio → 60:20:20	Acc 88.1% (for the lightweight models)
Wegner et al. (25)	2022	17	VGG 13	Non congenital model: 14,035 echocardiograms (five-fold cross-validation) Congenital Model: Train: 139,910 frames Test: 35,614 frames	Non-congenital model: Acc 48.3% in patients with C/SHD and 66.7% in patients with C/SHD Congenital model: Acc 76.1%
Zamzmi et al. (18)	2019	15	VGG	Train: 70 patients Testing: 30 patients	Acc 91.6%
Zhang et al. (26)	2018	23	DCNN	Training/Validation Dataset: 7168 individually labeled videos (5-fold cross-validation)	Acc 84%

Acc, accuracy; AUC, area under the curve.

### 3.3. Probe guidance

As previously mentioned, in echocardiography the operator must gather images at several standardized viewpoints. These echocardiographic views are acquired by scanning the patient while positioning the transducer at particular angles and positions with respect to the heart. These clinical procedures require specialized personnel to manually navigate the probe towards the correct imaging plane, which can be challenging for a novice operator (41). Moreover, the sonographers’ intense workload has put them at risk for health problems such work-related musculoskeletal illnesses (42). To lessen the heavy workload of sonographers, speed up examinations, and produce high-quality, standardized, and operator-independent imaging results, the automation of the US scanning process holds considerable promise.

Additionally, an assisted probe guidance system has the potential of improving access to care in remote or rural communities by reducing the level of necessary expertise (43). POCUS in particular is envisioned to be operated by inexperienced users who may have not received any formal training, as such an automated system could reduce the risk of non-diagnostic and misleading imaging (44, 45). However, the interpretation of the extremely complex and changeable US images acquired throughout the scan as well as their spatial correlations makes autonomous probe guidance towards the typical scan planes a difficult challenge (43).

Recently, several authors have attempted to develop an automated probe guidance system for scanning US images (43, 46–48). In this regard, Milletari et al. (44) presented a reinforcement learning strategy *via* a Deep Q-Network, to provide instructions (9 supported actions) to scan the LV through the PLAX cardiac window. Their approach is trained using 22 different simulated US acquisition environments (corresponding to approximately 160,000 US images) and tested on 5 distinct environments with about 40,000 scans each. The authors also train a classifier to learn from the same data in a fully supervised manner as a mean of comparison. Their approach correctly guided the user in 86.1% of the cases in the test set, while the classifier obtained a correct prediction in 77.8% of the data.

On the other hand, Gu et al. (41) used view conversion, US image characteristics and anatomical knowledge to anticipate what an unseen view might resemble as the user moves the transducer. Based on a learned A4C-to-A2C mapping, the team suggested a novel constrained conditional GAN to produce geometrically accurate and aesthetically pleasing A2C images. They utilized the 450 pairs of A4C/A2C echo cines synced by heart rhythm from the public CAMUS challenge dataset. The segmentation mask area of the produced and ground-truth images exhibits an 84% correlation in their quantitative analysis of the images, proving the framework’s capacity to make reliable predictions for a variety of cardiac shapes.

Commercial solutions for probe guidance are also available, notably Caption Health, which uses DL technology to give beginner users real-time turn-by-turn instructions and thereby collect TTE images (45). The software, recently approved by the US Food and Drug Administration, uses several connected DL algorithms to make 3 estimates simultaneously: (1) the diagnostic quality; (2) the distance between the current probe location and the location anticipated to optimize the image; and (3) corrective probe manipulations to enhance diagnostic quality. More than 5 million observations from 15 registered sonographers were used to train these algorithms. However, details regarding the methodological implementation are not publicly available, hindering development in this area. The same software was later tested in (49), involving 19 first-year medical students who had no prior experience with US scanning patients. In the PLAX, A4C, and A2C, the novices acquired diagnostic-quality images in 58, 86, and 68% patients, respectively.

The field of assisted probe navigation in echocardiography is still in its early stages. Nevertheless, the current research shows great promise. Automated probe guidance can be addressed using different strategies, as reflected on the studies included in this review. The most consensual proposed solution is the prediction of a set of instructions in real-time to assist the user. One of the main obstacles to this approach is the lack of data, particularly the high volume of annotations from different experts required for the supervised models. To overcome this limitation, augmented solutions, e.g., echocardiography synthetization and simulated environments, could be used in future research.

In summary, AI guidance during data acquisition for the optimal angle, view, and measurements would make echocardiography less operator-dependent, overcoming human limitations of both distraction and fatigue, while standardizing data acquisition. Additionally, AI-assisted acquisition holds great promise on an educational level, since these systems can help beginner US operators build expertise, by recognizing off-axis acquisition and incorrect views, as well as provide guidance on how to move the probe and obtain diagnostic level echocardiograms. With the advance of POCUS technology, AI applied to echocardiography at point of care locations could increase the utility of these devices by non-experts in primary and emergency departments, as well as medically underserved areas.

## 4. Limitations in assisted echocardiographic acquisition

Although there is already significant and promising research on this subject, there are still limitations that will be explored in this chapter. Lack of large, widely accessible, and well-annotated echocardiographic datasets for neural network training is the biggest obstacle to advancement of AI applications in echocardiography. The majority of effective AI techniques are supervised, and their effectiveness relies on the meticulous labeling of input data, frequently involving technicians and cardiologists. Consequently, the performance of AI may be constrained by the intra- and inter-observer variability in data labeling. These databases must also contain sufficient “real-world” heterogeneity, reflecting the range of practice and imaging methods in the field, in order to apply AI into widespread clinical practice. As a result, in order to acquire generalization and boost the dependability of a suggested model, it is crucial to train and evaluate AI models on large multi-vendor and multi-center datasets. Recently, two initiatives to develop publicly available large echocardiographic databases, from Stanford University (50) and University of Lyon (12), have opened exciting opportunities. However, the data is not accompanied by relevant clinical patient data and outcomes, as well as limited by the number of available views (only A4C and A2C).

A potential method to overcome the limitation of having small training datasets would be augmenting the dataset with realistic transformations that could help improve generalizability of AI models. On the other hand, realistic transformations need to be used to genuinely simulate variations in cardiac US images and transformed images should not create artifacts. Alternatively, GANs, which include a generator and a discriminator model, are trained until the model generates images that are not separable by the discriminator and could be used to generate realistic echocardiograms. The introduction of these transformations during the training process could make AI models more robust to small perturbations in input data space. However, these techniques only mitigate the aforementioned limitations, and ultimately real data is always better since it has more variability.

Another challenge when facing echocardiographic datasets is the image quality. AI applications in echocardiography are notoriously challenging when compared to other medical imaging modalities, due to the nature of an echocardiographic exam, such as patient-specific variables (e.g., obesity and artifacts) and US speckle noise pattern. Hence, quality control and quality enhancement techniques are crucial to the clinical workflow, particularly for less experienced users.

Furthermore, AI applications in medical imaging are also limited by “black box” methods in ML and DL. Interpretability is of particular importance in medical imaging since the analysis of a medical examination inform medical decisions and could expose a patient to undue risk. Thus, machine errors will always need to be safeguarded by humans to ensure above all else to do no harm. In spite of the fact that the DL community has been actively researching the problem of making AI models transparent, much more research is still required in this field (51).

## 5. Conclusion

Fully automatic acquisition of echocardiograms has the potential of radically changing the workflow in clinical laboratories and more remote areas. The findings in this review demonstrate considerable improvements in image quality and cardiac view identification and suggest that DL approaches are promising to fully automated echocardiogram processes. However, some of the issues raised have yet to be resolved, and research is required to build public confidence in new technologies, backed by initiatives to create transparent and explainable models.

## Author contributions

SF and JP conceived of the study conception and design. SF collected the data, performed the analysis, drafted the manuscript and designed the figures. JP was involved in planning and supervising the work, aided in interpreting the results, and worked on the manuscript. JP and MC revised the article critically and approve the final version of the article to be published.

## Funding

This work was funded by National Funds through the FCT – Portuguese Foundation for Science and Technology within the scope of project THOR DSAIPA/AI/0083/2020 and LA/P/0063/2020.

## Conflict of interest

The authors declare that the research was conducted in the absence of any commercial or financial relationships that could be construed as a potential conflict of interest.

## Publisher’s note

All claims expressed in this article are solely those of the authors and do not necessarily represent those of their affiliated organizations, or those of the publisher, the editors and the reviewers. Any product that may be evaluated in this article, or claim that may be made by its manufacturer, is not guaranteed or endorsed by the publisher.
